# *FecX*^*Bar*^ a Novel *BMP15* mutation responsible for prolificacy and female sterility in Tunisian Barbarine Sheep

**DOI:** 10.1186/s12863-017-0510-x

**Published:** 2017-05-15

**Authors:** Narjess Lassoued, Zohra Benkhlil, Florent Woloszyn, Ahmed Rejeb, Mohamed Aouina, Mourad Rekik, Stephane Fabre, Sonia Bedhiaf-Romdhani

**Affiliations:** 1 Laboratoire des Productions Animales et Fourragères, INRA-Tunisie, Université de Carthage, El Menzah, 1004 Tunis, Tunisia; 2 GenPhySE, Université de Toulouse, INRA, INPT, INP-ENVT, Castanet Tolosan, France; 3 Ecole Nationale de Médecine Vétérinaire, Université de la Manouba, 2020 Sidi Thabet, Tunisia; 4 International Center for Agricultural Research in the Dry Areas (ICARDA), P.O. Box, 950764, Amman, 11195 Jordan

**Keywords:** Barbarine, *BMP15*, Major gene, Ovulation, Prolificacy, Sheep

## Abstract

**Background:**

Naturally occurring mutations in growth and differentiation factor 9 (*GDF9*) or bone morphogenetic protein 15 (*BMP15*) genes are associated with increased ovulation rate (OR) and litter size (LS) but also sterility. Observing the Tunisian Barbarine ewes of the “W” flock selected for improved prolificacy, we found prolific and infertile ewes with streaky ovaries. Blood genomic DNA was extracted from a subset of low-ovulating, prolific and infertile ewes of the “W” flock, and the entire coding sequences of *GDF9* and *BMP15* were sequenced.

**Results:**

We evidenced a novel polymorphism in the exon 1 of the *BMP15* gene associated with increased prolificacy and sterility. This novel mutation called *FecX* ^*Bar*^ is a composite polymorphism associating a single nucleotide substitution (c.301G > T), a 3 bp deletion (c.302_304delCTA) and a C insertion (c.310insC) in the ovine *BMP15* cDNA leading to a frame shift at protein position 101. Calculated in the “W” flock, the *FecX* ^*Bar*^ allele increased OR by 0.7 ova and LS by 0.3 lambs (*p = 0.08*). As for already identified mutations, homozygous females carrying *FecX* ^*Bar*^ exhibited streaky ovaries with a blockade at the primary stage of folliculogenesis as shown by histochemistry.

**Conclusions:**

Our investigation demonstrates a new mutation in the *BMP15* gene providing a valuable genetic tool to control fecundity in Tunisian Barbarine, usable for diffusion program into conventional flocks looking for prolificacy improvement.

## Background

In livestock species, especially in meat sheep, improvement of reproductive traits such as ovulation rate (OR) and embryo survival which are the main components of litter size (LS) or prolificacy is of high economic importance. Sheep husbandry is a main pillar of the red meat value chain in Tunisia and several breeding programs are being implemented to sustain genetic improvement of sheep meat production retaining traits as diverse as meat quality, growth rate, environmental adaptation and prolificacy [1]. Considering the latter trait, most of the sheep breeds and strains in Tunisia are low prolific. Thus, a prolificacy-based selection program was implemented since 1979 by the Tunisian National Institute for Agronomic Research (INRAT) in the experimental center of Oueslatia Kairouan by screening prolific ewes among the fat tailed Barbarine sheep which represents over 60% of the national sheep population. The selection process yielded the prolific “W” strain with LS of 1.6 and a fertility rate ranging between 82 and 91% [2]. LS in conventional flocks of the same breed is about 1.1 [3]. During the 1999–2014 period, repeatability and heritability estimates of prolificacy in the “W” flock were calculated to be 0.62 and 0.13, respectively [3]. Compared to an average OR of 1.1 in the normal Barbarine ewe, the average OR of the “W” females was 1.9 with an upper individual mean OR reaching 3.1 [4]. However, a number of infertile females were recorded in the “W” flock affecting global fertility and examination using laparoscopy of the ovaries of these females revealed their streak aspect similar to what has been reported by [5] and most likely indicative of permanent sterility.

Interestingly, numerous studies in sheep identified point mutations in genes of the bone morphogenetic protein (BMP) family of growth factors associated to increased prolificacy but also to sterility [6] resembling the phenotypes observed in the Barbarine “W” flock. Indeed, up to now, 6 different mutations were evidenced in the bone morphogenetic protein 15 (*BMP15*) gene and 3 mutations in the growth and differentiation factor 9 (*GDF9*) gene responsible for this prolific and sterile double phenotype. The ovine *BMP15* gene is carried by the X chromosome and is also known as *FecX* (Fecundity X gene). The 6 *FecX/BMP15* mutated alleles were identified in various ovine breeds: *FecX* ^*I/*^ *BMP15* ^*c893T>A*^ and *FecX* ^*H/*^ *BMP15* ^*c871C>T*^ in New Zealand Romney [7], *FecX* ^*G/*^ *BMP15* ^*c718C>T*^ and *FecX* ^*B/*^ *BMP15* ^*c1100G>T*^ in Irish Cambridge and Belclare, *FecX* ^*L/*^ *BMP15* ^*c962G>*A^ in French Lacaune [8] and *FecX* ^*R/*^ *BMP15* ^*c455_471del*^ in Spanish Rasa Aragonesa [9, 10]. Concerning the *GDF9* or *FecG* gene on the ovine chromosome 5, one mutated allele was identified in Cambridge and Belclare sheep and is known as *FecG* ^*H*^ */GDF9* ^*c1184C>T*^ [11]. A second mutation was evidenced in Icelandic sheep as *FecG* ^*T*^ */GDF9* ^*c1279A>C*^ [12] and more recently, a third mutation was discovered in Ile-de-France sheep reared in Brazil and named *FecG* ^*V*^ */GDF9* ^*c943C>T*^ [5]. For both *BMP15* and *GDF9*, all these variants affect the open reading frame. They are considered as loss-of-function mutations increasing OR and thus prolificacy at the heterozygous state, but sterility by blockage or deep alteration of the ovarian follicular development at the homozygous state [6, 13].

Other prolific mutations in *BMP15* and *GDF9* genes segregate in ovine populations but were never associated to sterility; homozygous being more prolific than heterozygous carriers. This is evidenced for*FecX* ^*Gr*^ */BMP15* ^*c950C>T*^ and *FecX* ^*O*^ */BMP15* ^*c1009C>T*^ in the French Grivette and Polish Olkuska breeds, respectively [14]; also *FecG* ^*E*^ */GDF9* ^*c1034T>G*^ in Brasilian Santa Ines and *FecG* ^*NW*^ */GDF9* ^*c1111G>A*^ in Norwegian White sheep [15, 16]. Such dose-dependent prolific mutations were initially identified in the BMP receptor type 1B gene (*BMPR1B*, also known as *FecB*) in the Booroola sheep [17–19] and more recently in the beta-1,4-N-acetyl-galactosaminyl transferase 2 (*B4GALNT2/FecL*) gene in Lacaune sheep [20]. With the identification of several causal mutations affecting prolificacy in sheep, numerous ovine prolific breeds were genotyped for these known polymorphisms. Davis et al. originated the *FecB/BMPRIB* Booroola mutation (*FecB* ^*B*^) in Garole sheep of India [21]. Thereafter, *FecB* ^*B*^ was found naturally segregating in many Chinese breeds such as Hu, Han, Huyang, Cele, Duolang and Bayanbulak [22–24], in Indian Bonpala [25] and in Iranian Kalehkoohi sheep [26]. Some of these known variants in *BMP15* and *GDF9* were also found in various populations. *GDF9* ^*c1111G>A*^ (also known as G7 polymorphism) primarily detected in Belclare is associated to increased prolificacy in Norwegian White sheep [11, 16, 27]. The origin of *FecG* ^*H*^ */GDF9* ^*c1184C>T*^ and *FecX* ^*B/*^ *BMP15* ^*c1100G>T*^ was established in the Lleyn breed coming from the Lleyn peninsula of Wales [28]. Moreover, Belclare and Cambridge mutation *FecX* ^*G/*^ *BMP15* ^*c718C>T*^ is surprisingly present in Chinese small tail Han ewes [29]. The search for these known mutations in *BMPR1B*, *BMP15* and *GDF9* by specific genotyping failed in five sheep breeds reared in Tunisia; these are Barbarine, Queue Fine de L’Ouest, Noire de Thibar, Sicilo-Sarde and D’man breeds [30]. However, a second study identified the segregation of the *FecX* ^*B/*^ *BMP15* ^*c1100G>T*^ allele in prolific Barbarine ewes reared in conventional flocks, with an allele frequency of 21% [31]. Using the same PCR-RFLP technique for the polymorphism *FecG* ^*H*^ */GDF9* ^*c1184C>T*^ in the *GDF9* gene, some reports highlighted the existence of the mutation in Barbarine animals, 10 years ago, both in conventional [32] and in the “W” flocks [33]. The prolific and sterile double phenotype documented in ewes of the Barbarine “W” flock fits very well with the segregation of such mutations in the *BMP15* and *GDF9* genes, however, by sequencing recently the entire *BMP15* and *GDF9* coding sequences of selected high-ovulating, low-ovulating and infertile “W” ewes, *FecX* ^*B*^ and *FecG* ^*H*^ alleles were absent. The objective was to characterize of what could be a new mutation in the *BMP15* gene affecting the ovarian function of Barbarine sheep.

## Methods

### Animal material

The “W” strain line was originally created using prolific Barbarine ewes purchased from the Bureau of Livestock and Pastures (Office de l’Elevage et des Pâturages (OEP)) flocks present in the Zaghouan governorate, in North of Tunisia under semi-arid conditions. The nucleus of the prolific line was then reared at INRAT Ouesslatia research station. In 1979, the size of the flock reached 65 ewes and 7 rams. Reproduction was managed through 7 families. For many years, one ram and its replacement were assigned for each family. For mating, females born in a given family were systematically moved to another family to limit inbreeding and replacement animals were selected based on their litter size records (LS) [3]. Regarding to particular circumstances that have arisen in the last decade, the genetic monitoring of the flock was disturbed followed by a severe reduction in the flock size.

OR of females of the “W” flock was observed by laparoscopy during one or several breeding seasons. For the gene sequencing study and initial polymorphism discovery, 25 ewes were selected based on OR records (records between 2010 and 2014) and then classified as low-ovulating (*n* = 7), high-ovulating (*n* = 11) and infertile ewes exhibiting streak ovaries (*n* = 7). The low-high ovulating cut-off classification was set at individual mean OR = 1.66 to distinguish between individuals with typically one ovulation per estrus cycle (occasionally two) and individuals with typically two or more ovulations per estrus cycle but allowing an occasional lower value. Servicing rams of the “W” flock (*n* = 17) were analyzed by genotyping and sequencing. Thereafter, all females and males of the “W” flock present in 2015 were specifically genotyped for the identified mutation (*n* = 101).

A second genotyping analysis was conducted on 137 Barbarine animals, non-selected for prolificacy, originating from conventional flocks belonging to several state and research farms. All procedures were approved by the Agricultural and Scientific Research Government Committees (CPERA-Tunisia) in accordance with the guidelines for the Care and Use of Agricultural Animals in Agricultural Research and Teaching.

### Blood samples and DNA extraction

Approximately 5 ml blood was collected aseptically by venipuncture from the jugular vein using vacutainer collection tubes containing EDTA. All samples were then immediately refrigerated and transported to the laboratory under low temperature. The genomic DNA was extracted from white blood cells following a salt-based DNA extraction [34]. Genomic DNA samples were stored at −20 °C for further analysis.

### DNA sequencing analysis

Entire ovine *BMP15* and *GDF9* genes (from exon 1 to exon 2) were amplified for the 25 selected ewes by long-range PCR (Fermentas, Life Technologies) using primers designed by extracting reference genomic sequences (Oar_v3.1) from ovine chromosome X (NC_019484) and chromosome 5 (NC_019462), respectively. As a first step, forward 5′-TTCCTTGCCCTATCCTTTGTG-3′ and reverse 5′- TCTTCACCCCAAACCGTCTA-3′ primers were used to amplify the ovine *BMP15* gene, and forward 5′-TCGGACGGACTAAGAGTAGAAGA-3′ and reverse 5′-GGTTTGCCAGGTAAGAACAC-3′ primers were used to amplify the ovine *GDF9* gene. Long-range amplification primers and internal primers (Table 1) were used for Sanger sequencing reaction realized via the Big Dye Terminator v3.1 Cycle Sequencing Kit and analyzed on an ABI3730 sequencing machine (Applied Biosystems).

**Table 1:** Primers used for long-range PCR, sequencing and genotyping of ovine *BMP15* and *GDF9* genes

Gene	Primer sequence (5′→3′)	Location	Method
*BMP15*	TTCCTTGCCCTATCCTTTGTG	exon 1	long-range PCR/seq.
*BMP15*	GAGGCCTTGCTACACTAGCC	exon 1	genotyping*FecX* ^*+*^
*BMP15*	TGAGAGGCCTTGGCTACACA	exon 1	genotyping*FecX* ^*Bar*^
*BMP15*	ACTTTTCTTCCCCATTTTTCTGC	exon 1	sequencing
*BMP15*	CGCTTTGCTCTTGTTCCCTC	exon 2	sequencing
*BMP15*	GGCACTTCATCATTGGACACT	exon 2	sequencing
*BMP15*	GGCAATCATACCCTCATACTCC	exon 2	sequencing
*BMP15*	TCTTCACCCCAAACCGTCTA	exon 2	long-range PCR/seq.
*GDF9*	GAAGACTGGTATGGGGAAATG	exon 1	long-range PCR/seq.
*GDF9*	CCAATCTGCTCCTACACACCT	exon 1	sequencing
*GDF9*	TGGCATTACTGTTGGATTGTTTT	exon 2	sequencing
*GDF9*	TGAACGACACAAGTGCTCAGG	exon 2	sequencing
*GDF9*	GATAGCCCTCTCTTCTGGTCA	exon 2	sequencing
*GDF9*	GCTCCTCCTTACACAACACACAG	exon 2	sequencing
*GDF9*	GTGTTCTTACCTGGCAAACC	exon 2	long-range PCR/seq.

### Polymorphism genotyping

**Fig. 1:**
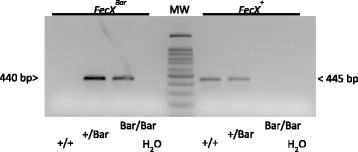
Allele-specific PCR amplification at the *FecX* ^*Bar*^ locus.*FecX* ^*Bar*^and*FecX* ^*+*^allele- specific PCR amplification of genomic DNA from Barbarinenon-carrier ewes (+/+), heterozygous (+/Bar) and homozygous carrier (Bar/Bar) of the *FecX* ^*Bar*^allele. Amplified bands were resolved on a 1% agarose gel (MW: molecular weight marker). H20 was used instead of genomic DNA as a contamination PCR control

*FecX* ^*B*^ and *FecG* ^*H*^ allele genotyping was performed through Restriction Fragment Length Polymorphism (RFLP) approach as previously described [11]. Restriction enzyme digestion by DdeI (New England Biolabs) of each specific PCR product was performed overnight at 37 °C to avoid for partial restriction. A genotyping test by allele specific PCR amplification was established for the detection of the *FecX* ^*Bar*^ mutation in the W flock and other non-selected Barbarine flocks (conventional). For each individual, a fragment of 445 (or 440) bp from *BMP15* exon 1 is amplified by two independent PCR using the same forward primer 5′-TTCCTTGCCCTATCCTTTGTG-3′ and one of the two allele specific reverse primer**,** 5′-GAGGCCTTGCTACACTAGCC-3′ for the *FecX* ^*+*^ wild-type allele and 5′-TGAGAGGCCTTGGCTACACA-3′ for the *FecX* ^*Bar*^ mutated allele. After 35 cycles of amplification (94 °C/30s, 62 °C/30s, 72 °C/30s), the PCR products were analyzed on 1% agarose gel. Heterozygous ewes were positive for the two PCR while homozygous ewes were positive only for one PCR depending on the carried allele (Fig. 1). The *BMP15* gene is located on the X chromosome, thus rams were expected to be positive only for one of the two PCR’s.

### Histological analysis

Four adult ewes from the “W” flock genotyped as non-carriers (*n* = 2) or *FecX* ^*Bar*^ homozygous carriers (*n* = 2) were unilaterally ovariectomized under local anesthesia with parenteral subcutaneous infiltration of 2% lidocaine. After recovery, ovaries from the two cyclic*+/+* ewes (presence of corpora lutea) and the two sterile *Bar/Bar* ewes bearing streak ovaries were fixed in 4% formaldehyde solution for 48 h and embedded in paraffin. The paraffin-embedded tissues were sectioned serially at 3 μm and these sections were stained with hematoxylin and eosin solution for light microscopy studies as described [35]. Different types of follicles, primordial follicles, primary, secondary or pre-antral, antral or tertiary and ovulatory follicles were identified as described [36].

### Statistical analysis

For the genotype-OR association among the 25 selected ewes, the genotype effect at *GDF9* or *BMP15* locus was tested by one-way ANOVA followed by a Tukey multiple comparison test. For the population study, LS at birth was defined as the total number of lambs born dead or alive per ewe lambing. Statistical analysis using least-squares techniques was performed to assess non genetic effects to be included in the final model [37]. The model applied included fixed effects of combined season-year of lambing [S*Y_*j*_], lambing interval nested within parity [*Hiera*_*kl*_] that corresponds to the age of the ewe when the ewe lambs for the first time, and to the interval between two consecutive parturitions for subsequent lambing.

$$ {Y}_{ijklm}=\mu + S\ast {Y}_j+{Hiera}_{kl}+{e}_{j klm} $$

Only significant fixed effects at the probability level of 5% were then considered to adjust the litter size performance (Y_jklm_) and it was for *Hiera* effect. The litter size performance was adjusted to the *Hiera* factor using the following equation [38]:

$$ {y_{ikl}}^{\ast }={\overset{-}{y}}_{kl}+\frac{y_{ikl}-{\overset{-}{y}}_{kl}}{\delta_{kl}}\times {\sigma}_b $$

Where ${y_{ikl}}^{*}$ is the litter size performance (LS) of the i^th^ ewe in the kl^th^ Hiera factor, *δ*_kl_ is the standard deviation of the error by lambing interval nested within parity, σb is the global residual deviation, and ${\overset{-}{\mathrm{Y}}}_{\mathrm{kl}}$ corresponds to the litter size average. The GLM procedure and Duncan’s multiple range test were used for LS and OR comparison within genotype groups.

## Results

### BMP15 and GDF9 sequence analysis

**Fig. 2:**
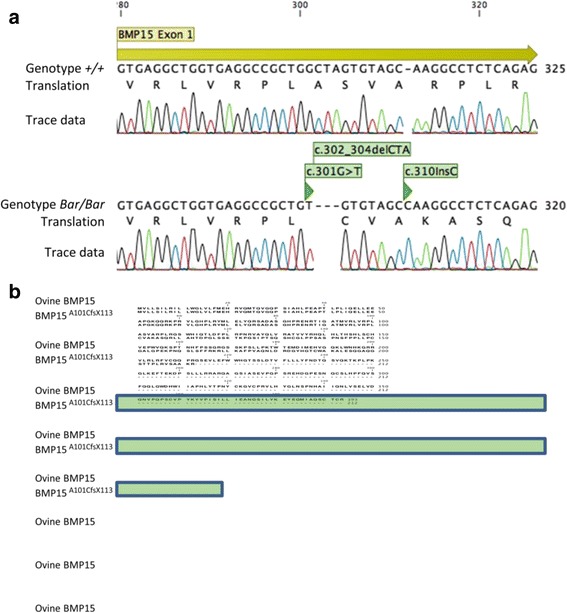
Sequencing of homozygous ewes carriers and non-carriers of the *FecX* ^*Bar*^ allele. **a** Sanger sequencing of the end of first exon of the ovine *BMP15* gene. Nucleotide numbering and amino-acid translation are relative to the ATG start site. The *FecX* ^*Bar*^ variant haplotype (*Bar/Bar*) is indicated within* green boxes* and compared to wild-type haplotype (*+/+*). **b** Protein alignment between reference ovine BMP15 and Barbarine mutated BMP15^A101CfsX113^ translated from homozygous *FecX* ^*Bar*^ variant. The 112 alternative amino-acids generated by the frameshift (fs) are highlighted in *green*. Views were obtained from CLC MainWorkbench 7.6.4 (Qiagen Aarhus)

Following laparoscopic observation of Tunisian Barbarine ewes of the “W” flock selected for increased LS, ewes were classified in 3 phenotypic groups regarding OR: low-ovulating (*n* = 7), high-ovulating (*n* = 11) and “streaky-bearing ovaries” females (*n* = 7). Animals in the latter class were kept in the flock for experimental purposes (Table 2). Surprisingly, among these 25 selected ewes, the *FecX* ^*B*^ allele in *BMP15* and the *FecG* ^*H*^ allele in *GDF9* were evidenced in any of the sequences. In line with what has been reported for Cambridge and Belclare sheep [11], we found the already described G3, G5 and G6 polymorphisms within the *GDF9* gene with no significant genotype association with the prolific or sterile phenotypes. In striking contrast, apart from the c.28_30delCTT already known as a non-causal polymorphism [11; 14], we discovered an original haplotype of 3 polymorphisms lying on 10 bp within the exon 1 of *BMP15* (Fig. 2a). This local haplotype associated the single nucleotide substitution c.301G > T, the 3 bp deletion c.302_304delCTA and the C nucleotide insertion c.310insC. The polymorphism nomenclature [301G > T; 302_304delCTA; 310insC] was given according to the ovine *BMP15* cDNA [NM_001114767]. As shown in Table 2, the mutated haplotype appeared homozygous in all “streaky ovaries” ewes, heterozygous in 9 of the 11 high-ovulating ewes, but absent from low-ovulating ewes. When grouped by genotype, the mean OR of heterozygous ewes was significantly higher than for non-carrier or homozygous carrier ewes (2.42 ± 0.54, *n* = 9 vs. 1.34 ± 0.49, *n* = 9 vs. 0 ± 0, *n* = 7; *P* < 0.0001, mean ± SD) fitting very well with a causal mutation. This novel mutation was named *FecX* ^*Bar*^ (Barbarine prolific allele of the *FecX/BMP15* gene) in accordance with the current names for existing fecundity genes. At the protein level, the *FecX* ^*Bar*^ mutation alters BMP15 by a non-conservative substitution (Ala > Cys) at position 101 followed by a frame shift coding for 112 alternative amino-acids terminated by a stop codon at position 113 of the alternative sequence (p.Ala101CysfsTer113, Fig. 2b). The additional alternative amino acids did not contain any putative conserved functional domains.

**Table 2:** *BMP15* genotypes of 25 selected ewes from the Barbarine “W” flock with different ovulatory phenotypes

Ewe ID	Phenotype group	OR mean	OR1	OR2	OR3	c.28_30delCTT	c.301_310 locus
8136	low-ovulating	1.0	1	1		del/del	+/+
8163		1.0	1	1	1	+/+	+/+
8218		1.0	1	1	1	+/+	+/+
8219		1.0	1	1		+/del	+/+
8195		1.3	1	1	2	+/del	+/+
8237		1.3	1	1	2	+/+	+/+
8247		1.3	1	1	2	+/del	+/+
8193		1.7	1	2	2	+/+	+/+
8146	high-ovulating	2.0	3	1		del/del	+/Bar
8182		2.0	2	2		del/del	+/Bar
8183		2.0	2	2	2	del/del	+/Bar
8189		2.0	2	2	2	del/del	+/Bar
8196		2.0	2	2	2	del/del	+/Bar
8159		2.5	3	2		+/+	+/+
8226		2.5	3	2		+/del	+/Bar
8149		3.0	3	3		+/del	+/Bar
8167		3.0	3			del/del	+/Bar
8249		3.3	3	3	4	del/del	+/Bar
8141	Streakyovaries	0				del/del	Bar/Bar
8166		0				del/del	Bar/Bar
8170		0				del/del	Bar/Bar
8171		0				del/del	Bar/Bar
8202		0				del/del	Bar/Bar
8228		0				del/del	Bar/Bar
8256		0				del/del	Bar/Bar

*ID* restricted animal identification number, *OR* ovulation rate; locus numbering relative to the ovine BMP15 cDNA (NM_001114767)

### *FecX*^*Bar*^ genotyping of Barbarine sheep populations

In order to search for the segregation of the *FecX* ^*Bar*^ allele in a larger population of Barbarine sheep, we developed an allele-specific PCR genotyping (Fig. 1). Firstly, the 25 sequenced ewes were analyzed by this PCR test indicating a 100% accuracy of the genotyping method. Thereafter, 17 servicing “W” rams were also genotyped by PCR and the genotype was 100% certified by Sanger sequencing. The *FecX* ^*Bar*^ mutation was searched by allele-specific PCR genotyping along with RFLP for *FecX* ^*B*^ and *FecG* ^*H*^ by in the actual “W” flock in 101 animals present in 2015 (74 ewes and 27 rams) and in 137 Barbarine animals (29 ewes and 108 rams) from several conventional flocks across Tunisia. *FecX* ^*B*^ and *FecG* ^*H*^ were absent from the 248 tested animals. For prolificacy trait, the low-high litter size cut-off classification was set at flock average LS = 1.12 to distinguish between individuals with simple litter size and individuals with typically more than one lamb per lambing. The frequency of the *FecX* ^*Bar*^ carrier females in the “W” flock reached 0.42 (39% of heterozygous and 3% of homozygous carrier) corresponding to 57% of heterozygous “W” ewes with a litter size exceeding 1.12 and a total of 86% of non-carrier homozygous ewes having a litter size less than 1.12 (Table 3). The value of GF by LS for heterozygous “W” showed that the Barbarine ewes, raised under low input production system, found difficulties to exteriorize its potential.

**Table 3:** Genotype frequency of females in two Barbarine ecotypes

		Flock “W”			Conv-flock	
Genotype	*n*	GF	GF by LS	*n*	GF	GF by LS
*FecX* ^*+*^ */FecX* ^*+*^	43	0.58	86% with LS < 1.12	29	1.00	88% with LS < 1.12
*FecX* ^*+*^ */FecX* ^*Bar*^	29	0.39	57% with LS > =1.12	−	0.00	
*FecX* ^*Bar*^ */ FecX* ^*Bar*^	2	0.03	−	−	0.00	−

*Conv-flock* Conventional flock (OEP; OTD), *GF* Genotype frequency

In the males of the “W” flock, the *FecX* ^*Bar*^ */Y* genotype frequency reached 0.37 (Table 4). A total of 80% of non-carrier “W” males had a litter size more than 1.12, which could be an inheritance of the *FecX* ^*Bar*^ allele from the dam.

**Table 4:** Genotype frequency of males in two Barbarine ecotypes

	Flock“W”			Conv-flock		
Genotypes	*n*	GF	GF by LS	*N*	GF	GF by LS
*FecX* ^*+*^ */Y*	17	0.63	80% with LS > 1.12	107	0.99	100% with LS < 1.12
*FecX* ^*Bar*^ */Y*	10	0.37	87% with LS > =1.12	1	0.01	−

*Conv-flock* Conventional flock (OEP; OTD), *GF* Genotype frequency

In contrast, the FecXBar allele was not found in the tested natural non-selected Barbarine females, except one male is carrier of this mutated allele. In conventional flocks, a total of 88% of females had a litter size less than 1.12.

### In vivo effects of the *FecX*^*Bar*^ mutation

Our primary sequencing analysis with selected “W” ewes indicated a positive relationship between *FecX* ^*Bar*^ mutation in *BMP15* and ovulation rate in Barbarine breed. After genotyping of the entire “W” flock, it was possible to associate the genotype to LS and OR for a sample of ewes of the flock (Table 5). The standardized litter size performance (LS) of ewes with *FecX* ^*+*^ */FecX* ^*Bar*^ genotype averaged 1.43 ± 0.51. This value tended to be higher than LS of non-carrier ewes (1.13 ± 0.32; *P* = 0.08). The same tendency was observed for OR trait (2.04 ± 0.75 vs. 1.37 ± 0.53, *P* = 0.08). By comparison, LS of wild-type ewes in conventional flocks was 1.08 ± 0.23 and thus not different from wild-type “W” females.

**Table 5:** Association of BMP15 genotypes with standardized litter size (LS) and ovulation rate (OR) in two Barbarine ecotypes

Ecotypes	Genotypes	*n*	OR (mean ± SD)	LS (mean ± SD)
“W” flock	*FecX* ^*+*^ */FecX* ^*+*^	*26*	1.37 ± 0.53^a^	1.12 ± 0.32^a^
	*FecX* ^*+*^ */FecX* ^*Bar*^	*19*	2.04 ± 0.75^a^	1.43 ± 0.51^a^
	*FecX* ^*Bar*^ */FecX* ^*Bar*^	*1*	1^a^	
Conventional flock	*FecX* ^*+*^ */FecX* ^*+*^		1.1	1.08 ± 0.23

^*a*^ *Difference* at *P* = 0.08

**Fig. 3:**
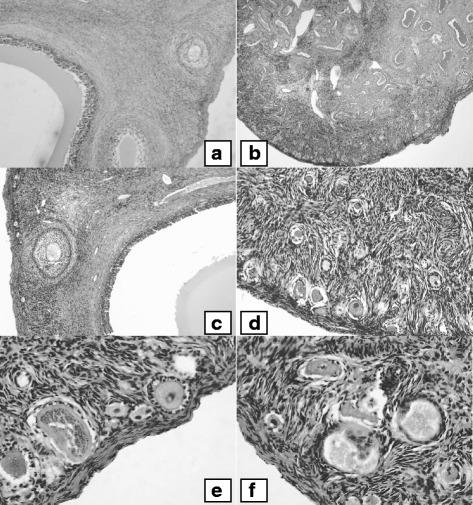
Histological sections of Barbarine sheep ovaries. Photomicrographs of histological section of the *FecX* ^*Bar*^non-carrier (**a** and **c**) or homozygous carrier ovaries (**b** and **d–f**). **a,c**, Evidence of follicular growth with the presence of secondary and tertiary follicles in normal ovaries. **b**, *FecX* ^*Bar*^/*FecX* ^*Bar*^ ovaries densely packed with primordial follicles in the cortex with no visible secondary or tertiary follicles. **d, e, f**, Ovarian cortex of *FecX* ^*Bar*^/*FecX* ^*Bar*^ovaries with primordial follicles and numerous abnormal follicular structures: primary-like follicles exhibiting enlarged oocytes with thickened zona pellucida surrounded by disorganized granulosa cell layers, and oocyte-free follicular structures

In parallel with the effect on OR and LS, it was interesting to investigate the streaky ovary phenotype repeatedly exhibited by adult females of the “W” flock coded as infertile and thereafter genotyped as homozygous carriers of the *FecX* ^*Bar*^ allele (Table 2). *FecX* ^*Bar*^ infertile ewes were characterized by an infantile genital tract and the ovaries did not carry any obvious follicular structures (Fig. 3). In contrast to non-carrier ovaries containing all follicle size classes and corpora lutea (Fig. 3a and c), histological studies of the *FecX* ^*Bar*^/*FecX* ^*Bar*^ ovaries revealed only primordial and primary follicles (Fig. 3b). Like ewes homozygous for previously described *BMP15* mutations, the cortical region of the *FecX* ^*Bar*^/*FecX* ^*Bar*^ ovaries was densely colonized by primordial and primary follicles (Fig. 3d). Many of these primary follicles were abnormally constituted of large oocytes with thickened zonapellucida surrounded by disorganized granulosa cell layers (Fig. 3e). Oocyte-free follicular structures were also observed (Fig. 3e and f).

## Discussion

Previous studies investigating inactivating mutations in *BMP15* and/or *GDF9* genes have shown that, while ewes heterozygous for the mutations have increased prolificacy, ewes homozygous for the mutation are sterile [6]. We had observed a similar phenotype, with both increased prolificacy and sterility in Tunisian Barbarian sheep of the “W” flock and thus investigated the presence of polymorphisms in *BMP15* and *GDF9* genes in this flock. Fitting well with the genetic determinism of this phenotype, previous preliminary studies using RFLP genotyping have evidenced the segregation of both *BMP15*/*FecX* ^*B*^ allele in high-prolific population of Barbarine ewe from conventional flocks [31] and *GDF9*/*FecG* ^*H*^ allele in both in conventional [32] and in the “W” flocks [33]. In the present study, none of the two alleles was found in the 248 Barbarine animals tested. Particularly for the “W” flock, this could be explained by a disturbed genetic management under the economic and political context in Tunisia during the last decade leading to elimination of important reproducers followed by a severe reduction of the flock size. This may have induced a bottleneck and the disappearance of some alleles.

However, a novel complex mutation in *BMP15*, *FecX* ^*Bar*^c.301–310 located at the end of the first exon, was observed to be present in the “W” flock, with ewes heterozygous for the mutation having increased ovulation rates and litter size whereas those homozygous for the mutation were sterile.

At the protein level, the *FecX* ^*Bar*^ mutation on DNA created a non-conservative substitution at position 101 followed by a frame shift coding for 112 alternative amino-acids. The new mutated protein sequence of 313 amino-acids is devoid of the *BMP15* mature domain (starting at position 269 in the normal protein) and does not carry any new functional or putative protein domain. Consequently, *BMP15* ^*A101CfsX113*^ is supposed to be a non functional protein as already described for a similar mutation creating a frame shift (*BMP15* ^*W154NfsX55*^) in Rasa Aragonesa sheep [9, 10] or mutations creating premature stop codon (*BMP15* ^*Q291X*^ and *BMP15* ^*Q239X*^) in Hanna and Cambridge sheep [7, 11]. Interestingly, all these latter mutations induced prolificacy at heterozygous state but sterility with streaky ovary phenotype when homozygous, resembling perfectly the phenotypes observed in Barbarine sheep of the “W” flock. Particularly, *FecX* ^*Bar*^ infertile ewes displayed an infantile genital tract and the ovaries did not carry any obvious follicular structures. As observed in Inverdale (*FecX* ^*I*^), Hanna (*FecX* ^*H*^) and Lacaune (*FecX* ^*L*^) homozygous ewes, the cortical region of the *FecX* ^*Bar*^/*FecX* ^*Bar*^ ovaries was densely colonized by primordial and primary follicles [7, 8, 39, 40]. All histological observations converge towards assuming a blockade of the primary to secondary follicle transition during folliculogenesis as an explanation of the infertility of the homozygous *FecX* ^*Bar*^ mutated Barbarine ewes.

By genotyping the entire “W” flock, we established that the *FecX* ^*Bar*^ allele segregates with a high frequency. Indeed, 42% of the female and 37% of the males were carrier of the *FecX* ^*Bar*^ allele perhaps indicating a particular selection pressure exerted on the *FecX* ^*Bar*^ allele to increase prolificacy of the “W” flock. In contrast, *FecX* ^*Bar*^ was not found in the tested non-selected Barbarine females from conventional flocks, except for one male. One would have expected more carriers since the “W” flock originates from this Barbarine population, but our DNA set from conventional flock may be too small and focused mainly on non prolific ewes. The presence of a ram carrying the *FecX* ^*Bar*^ mutation and belonging to a conventional flock could be the result of a progeny testing program for “W” rams conducted in 1999, where eight rams were transferred from INRAT to an OEP conventional flock [41]. A larger prospection into the Barbarine population, particularly on prolific females, would be necessary to find more *FecX* ^*Bar*^ carrier animals in order to conclude on the frequency of the mutation in conventional flocks.

Findings of this study point out that the *FecX* ^*Bar*^ allele causes increase of OR by +0.7 ova and LS by +0.3 lambs at each parturition. It is equivalent or slightly lower than for other already known mutations in *BMP15* acting on LS:*FecX* ^*R*^ increasing LS by +0.35 [10], and *FecX* ^*I*^ *, FecX* ^*H*^ and *FecX* ^*O*^ increasing LS by +0.6 [7, 14]. However, it should be noted that a few of the polyovulatory ewes present in the “W” flock were not carriers of the *FecX* ^*Bar*^ mutation. The factors increasing ovulation rate in these ewes are unknown, but could be related to other factors such as season, nutrition or as yet unidentified genetic factors [42]. Seasonal fluctuations were clearly observed in the “W” flock, as poly-ovulating ewes mated in spring/summer (May to July) had an OR of 1, whereas OR averaged 1.7–1.8 in autumn and winter [42].

## Conclusions

Our results evidenced a new mutation in ovine *BMP15* gene affecting the prolificacy and fertility of Barbarine ewes in the “W” flock selected for increased prolificacy. This new mutation named *FecX* ^*Bar*^ is to be added to the already-known 8 mutations in this major gene affecting prolificacy in sheep [13]. It is the first *FecX* mutation detected in the first exon of *BMP15*; all other mutations being in the second one.

This frame-shift mutation is supposed to be associated with the absence of BMP15 production by the oocyte, explaining the early blockage of folliculogenesis and the resulting streaky ovaries observed in infertile homozygous carrier females.

The presence at a high frequency of the *FecX* ^*Bar*^ carrier animals (females and males) in the “W” flock at INRAT represents an opportunity to disseminate this mutation in the conventional Barbarine population. Nevertheless, due to the sterility induced by crossing carrier animals, such genetic improvement will need strong and precise management. Moreover, the *FecX* ^*Bar*^ effect should also be analysis on other important traits such as lamb growth and survival, seasonality and global fertility to be fully usable in a genetic improvement program.

## Abbreviations

B4GALNT2: Beta-1,4-N-acetyl-galactosaminyl transferase 2
*BMP15*: Bone morphogenetic protein 15
Fec: Fecundity gene
*GDF9*: Growth and differentiation factor 9
LS: Litter size
OEP: Office de l’Elevage et des Pâturages
OR: Ovulation rate
OTD: Office des Terres Domaniales
